# Recommendations for ethical and responsible use of artificial intelligence in digital agriculture

**DOI:** 10.3389/frai.2022.884192

**Published:** 2022-07-29

**Authors:** Rozita Dara, Seyed Mehdi Hazrati Fard, Jasmin Kaur

**Affiliations:** School of Computer Science, University of Guelph, Guelph, ON, Canada

**Keywords:** ethical artificial intelligence, responsible innovation, transparency, accountability, fairness, trustable digital agriculture

## Abstract

Artificial intelligence (AI) applications are an integral and emerging component of digital agriculture. AI can help ensure sustainable production in agriculture by enhancing agricultural operations and decision-making. Recommendations about soil condition and pesticides or automatic devices for milking and apple picking are examples of AI applications in digital agriculture. Although AI offers many benefits in farming, AI systems may raise ethical issues and risks that should be assessed and proactively managed. Poor design and configuration of intelligent systems may impose harm and unintended consequences on digital agriculture. Invasion of farmers' privacy, damaging animal welfare due to robotic technologies, and lack of accountability for issues resulting from the use of AI tools are only some examples of ethical challenges in digital agriculture. This paper examines the ethical challenges of the use of AI in agriculture in six categories including fairness, transparency, accountability, sustainability, privacy, and robustness. This study further provides recommendations for agriculture technology providers (ATPs) and policymakers on how to proactively mitigate ethical issues that may arise from the use of AI in farming. These recommendations cover a wide range of ethical considerations, such as addressing farmers' privacy concerns, ensuring reliable AI performance, enhancing sustainability in AI systems, and reducing AI bias.

## Introduction

Digital agriculture refers to the use of advanced technologies that have transformed the agricultural sector by making farm operations more insightful and efficient. This can be achieved with the use of automated methods such as AI, Internet of Things (IoT), and collection and processing of farm data. Substantial amounts of data are collected and used by AI-based solutions in data-driven services and decision support systems (DSS) in farming applications (Zhongming et al., 2021). Farm data are combined with other data sources, such as weather data, to improve resource management and production (Sykuta, 2016).

The application of AI in agriculture and food has been on the rise. According to Statista's report, the use of AI-based technologies in the agriculture market has had significant growth. The Market is forecasted to grow from $1.1 billion in 2019 to more than $3.8 billion by 2024 (von See, 2022). AI solutions in agriculture refer to AI algorithms, software, and hardware (e.g., robots) used in farming applications (Ryan, 2022). AI software applications often analyze data to provide forecasts, recommendations, and assistance in decision-making to improve farm operations. Advanced hardware technologies used in farm robotics and automation such as robotic greenhouses, robotic harvesting, and milking robots in dairy farms have helped in increasing production yield.

**Figure 1:**
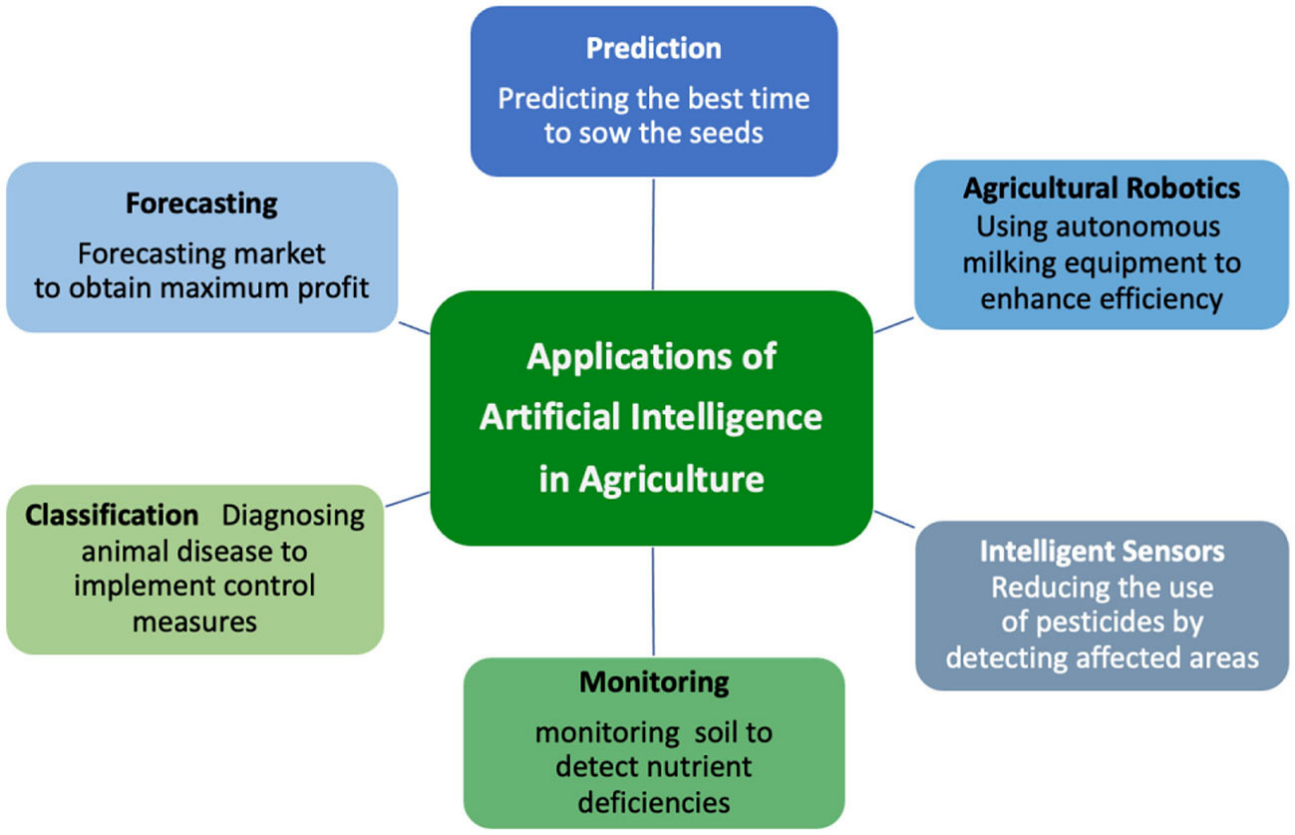
Artificial intelligence applications in digital agriculture.

Figure 1 illustrates AI applications in the agricultural system with some examples.

**Figure 2:**
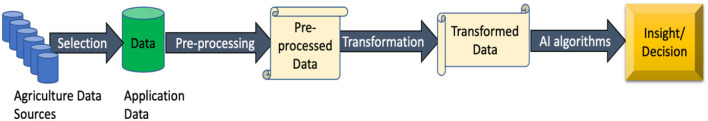
Knowledge discovery and information extraction process in artificial intelligence.

AI-based technology solutions for farming collect large volumes of data on various aspects of farming, such as production, environment, and machine data. Data from diverse sources such as wireless sensor networks, network-connected weather stations, monitoring cameras and drones, and historical information are used to build analytics tools for making more informed decisions or automated actions by farm robots (Sykuta, 2016). Collected data are preprocessed (e.g., cleaned) and transformed (e.g., integrated) into a uniform and appropriate format to be used by the AI algorithms. Finally, the AI algorithms are built with the collected data. After analysis, data are transformed into insight (Figure 2). Insight can be a pattern or trend analysis to help farmers make informed decisions or it could be automated operations performed by a machine or farming robots. For example, predictions can help farmers decide when to seed and harvest to approach the best productivity. In precision livestock farming, farmers can be informed about the possibility of disease or distress among animals on the farm. For instance, using image recognition tools plant disease can be detected or livestock animal behavior patterns can be monitored to help them with potential health issues (Ryan, 2022). Market demand and forecasting can help farmers adjust the type and amount of production and reach the best profitability.

Examples of AI-based agricultural tools include machines that are used on farms to hoe and harvest crops, perform weeding, drones to spray weeds and pesticides (Ryan et al., 2021b), and devices used in automatic milking (Shamshiriet et al., 2018). Robots have contributed to reducing the volume of chemicals sprayed on the crops by 80% (Revanth, 2019). This optimization has shown to decrease the expenditure on pesticides and herbicides by 90% and also to save the environment from the side effects of using chemicals (Revanth, 2019). Images collected from crops using drones can be used in diverse applications (Marvin et al., 2021), such as monitoring the status of soils for nutrient deficiencies, monitoring farm animal health, and crop cultivation optimization.

Although utilizing AI-based solutions in smart farming can provide numerous benefits for digital agriculture, they may raise ethical issues (der Burg et al., 2019). For example, access and use of large amounts of farm data can result in accurate and reliable AI models. However, it may make farmers concerned about the privacy and confidentiality of their data. Furthermore, the data collected from farms can raise concerns about data ownership i.e., who owns the farm data and who has control over the use of farm data (Mark, 2019). This can lead to a power imbalance between farmers and technology or service providers (Ryan et al., 2021a). Accountability is another issue when it comes to AI-technologies; for instance, if AI systems spray substantial amounts of water or pesticides due to errors in the system, who will be responsible for the loss of harvest? Automated tools' hardware such as harvesting robots may destroy plants during harvesting operations due to an error in the system or bad design.

This paper is aimed at examining ethical issues that may arise from the use of digital technologies on-farm and providing recommendations on how these issues can be mitigated. We first present an ethical AI framework in the context of digital agriculture. This framework captures ethical considerations using six principles: fairness, transparency, accountability, sustainability, privacy, and robustness. We then provide recommendations for ethical and responsible use of AI-based technologies in agriculture in the context of principles. A part of our recommendations is targeted at ATPs who are responsible for designing, developing, or governance of technologies used at farms. We also provide recommendations for policymakers who we believe are in a position to enforce and promote consideration of ethical requirements through policies. These recommendations can help in addressing the ethical issues in the digital agriculture domain and can help in bringing clarity in how the ethical design principles can be embedded to achieve responsible innovation in farming.

## Ethical framework for assessment of artificial intelligence-based solutions in agriculture

**Figure 3:**
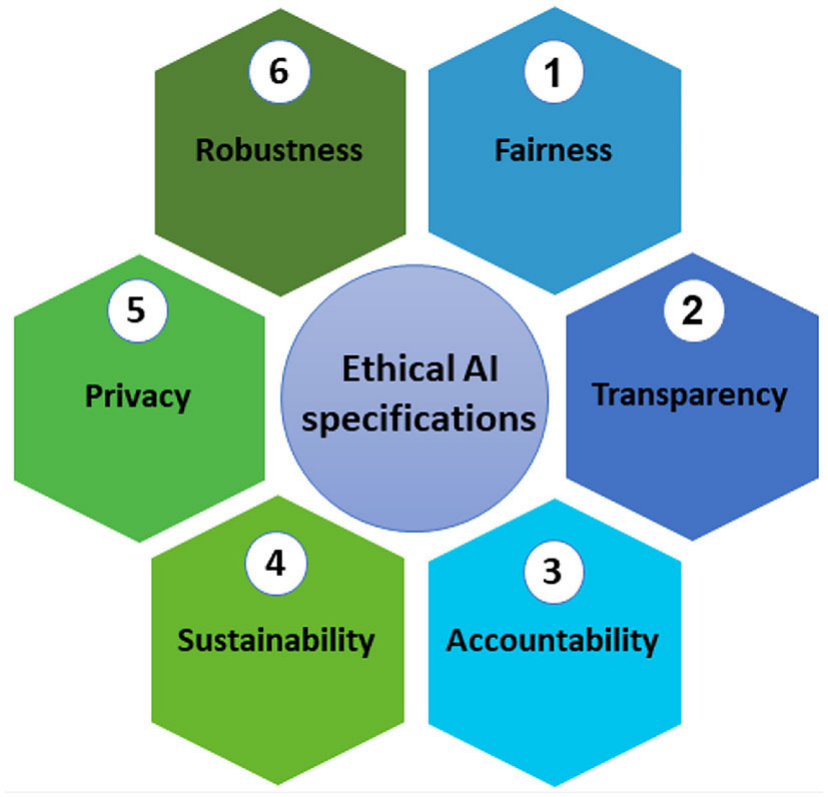
Ethical artificial intelligence principal in digital agriculture.

This section presents a framework for ethical AI. This framework sheds light on ethical considerations and issues that may arise from the use of AI-based solutions in farming. Studying the ethical framework can give us a lucid perspective on how to approach the design and development of AI-based technologies in digital agriculture. Building ethical technologies ensure ethical legitimacy and accountability and also help gain the trust of the stakeholders, in particular farmers, in the agricultural sector. Ethical principles are discussed in six categories (Figure 3).

Fairness is one of the principles of ethical AI. Fairness refers to monitoring and mitigating bias in the AI model, fair collection and use of data, and fair access to digital assets (Jobin et al., 2019). Bias in the AI model is the tendency to learn a preferred pattern of data rather than learn from the actual data distribution when the model is built. The constructed AI model will encounter bias if it has prejudiced assumptions made during the development process of algorithm or if there are prejudices in the data. Also, if the collected data is not an accurate representation of the context/problem, the AI model constructed from these data may encounter bias. Agriculture applications can be impacted by AI bias. For example, assume that an autonomous apple picking application is built to detect ripe apples. If the collected images for constructing AI model are largely provided from the red-type apples, the final model is biased toward these types of apples. This model may detect ripe green apples as raw apples in an orchard with green apples and may not pick them. Having diverse apple images of various types and stages of growth can lead to constructing a more reliable and inclusive model. Not only AI systems should be free of biases, they should also respect societal values such as human rights, democratic values, and diversity (OECD AI, 2019). Another unfair situation that may emerge from the use of AI systems in farming is imbalanced power distribution resulting from access and control of farm data and AI technologies. In cases where technology and services providers have access to data from a large number of farms and can control the AI systems, this power can be misused to control the market, share information with third parties, and override farmers rights to their own assets (Ryan, 2020). With proper adoption of fariness principle, all members in the community will benefit from AI tools fairly and inclusively (Aggarwal and Singh, 2021).

Transparency is another principle of ethical AI that reinforces the concept of trust. Transparency is a practice of being open about policies, actions, and laws with the stakeholders to enhance communication, common understanding, and collaboration. This is a core principle that exists in many ethical AI frameworks. For instance, the OECD transparency principle (Ryan, 2020) requires that individuals are informed about the data that are being collected from them, whether they are engaged in an automated AI-based decision making, and what decisions are made about them using their data. In the context of agriculture, lack of transparency can undermine farmers' trust and may result in their unwillingness to adopt the AI solutions or their discomfort to share farm data with the technology providers. The lack of interpretability and understandability of the AI model outcome also invades the transparency principle (Jobin et al., 2019). Interpretable AI refers to the degree to which humans can understand the results of the AI algorithms (Slack et al., 2019). Interpretable AI models should be developed in a way that can explain decisions to ordinary people. An interpretable AI model shows how decisions are made and what determining factors are considered for that decision (Ausloos and Veale, 2020). Interpretability with the aim of enhancing transparency of AI technologies is important for the development or governance of agricultural AI systems. For instance, to decrease the impact of greenhouse gases, AI tools can be used for the assessment and management of carbon dioxide in production facilities. Farmers should be able to challenge the given assessment of their carbon footprint, in cases where they think the decision was unfair or inaccurate.

Accountability is known as another key principle of ethical AI along with transparency (Dignum et al., 2018). The problem with available AI models in most automated decision-making systems is the lack of legal and policy transparency or clarity on who or which organization will be held accountable for the mismanagement, errors, or wrong decisions/recommendations made by the AI systems (van der Loeff et al., 2019). These are valid concerns that are associated with automated technologies used in farms. A case in point is where an AI system is designed to determine the optimal dose of fungicide and effective planning of fungicide application so that the farmers can enhance the yield and save money. However, if the fungicides are applied on fresh produce too late, this error may cause dire consequences for production and even consumers if traces of fungicides are left behind on harvested products. The ethical question that arises in this situation is who will be responsible for such errors? Without appropriate legal agreements that establish responsibilities and rights of actors involved in the development, maintenance, and use of these technologies, it will not be possible to identify who will be accountable for the errors, financial or reputational losses (Walter et al., 2017). Also, there has to be oversight by legal entities to ensure terms of use and data license agreements are ethical and the benefit of all parties are taken into consideration. Carolan (2020) explained an example of violating accountability by John Deere. This company has made farmers sign an agreement that the company has no responsibility if there is damage to the farmers' crops, land, profit, machines, or business. This raises an ethical dilemma on who will be responsible for farmers' financial loss if digital technologies fail to function properly. According to OECD (OECD AI, 2019), organizations developing, deploying, or operating AI systems in digital agriculture and related sectors should be accountable for the proper functioning of the tools and decisions.

Sustainable use and deployment of AI software and hardware need to be considered during the development and testing of AI-based technologies (Mark, 2019). Sustainability principle has several aspects. On the one hand, it requires that AI models are user friendly and useful for farmers (Vinuesa et al., 2020). For example, if an AI tool cannot provide actionable recommendations to help farmers, the system is not user friendly and may be defective in terms of design and usability. The usability of AI software is essential to make use of predictions and recommendations provided by the farm tool. Usefulness of AI-based technologies for farmers, on the other hand, depends on the accuracy and reliability of recommendations and actions. Software errors, hardware malfunction, and biased predictions are just some examples that impact sustainable deployment of AI solutions at farms. For instance, robots, drones, and autonomous/smart vehicles in farming may leak toxic chemicals and emit fumes which can lead to pollution, or apply too much or too little water on the farm due to errors or mismanagement of AI systems or algorithms (Kacary, 2021). Lastly, environmental sustainability should be taken into consideration when building an AI system in agriculture. Algorithms must be developed and measured in a way to reduce harm to the environment.

According to OECD (OECD AI, 2019), AI technologies should work safely, securely, and robustly during their farm operations. Robustness in farming technologies means that farmers have confidence in AI models and equipment due to their reliable performance and ability to secure the technologies from hacks and breaches. Generally, farms that rely on AI and technological automation are more vulnerable to hacking, sabotage, corporate espionage, or other failures that lead to reliability issues (Carolan, 2020). Reliability refers to the application of AI to predict when an asset (e.g., data, hardware, software) will fail, malfunction, or deteriorate. For instance, robots and automated decision-making tools in greenhouse horticulture have reduced the need for human intervention. This trend has also increased the need for reliable automated machines. Security breaches, weak AI models, low quality data, or usability issues that result in system failure to perform may damage farm production, result in misuse of farm resources, and lead to financial loss. Designing systems that can detect failures and self-perform construction will be needed in fully autonomous environments.

Ensuring data privacy and confidentiality are other ethical principles for AI systems. Privacy is referred to as the right of an individual to control the use, sharing, and retention of their personally identifiable information. Privacy is protected through regulations and legal agreements. Confidentiality on the other hand is ensured through legal agreements. The main concerns related to privacy include lack of control over their data, and issues such as what data are collected from farms, how they will be used, and with whom they may be shared (Mark, 2019). Stock et al. showed that 78% of farmers are concerned about their data being shared and sold by corporations without their consent or awareness (Stock and Gardezi, 2021). Farm data are highly vulnerable to misuse if they are shared with third parties. This is more of a concern when the data is shared without farmers' consent, or the data is processed or used beyond the stated purpose. Furthermore, farmers should be given a choice about their farm data collection, access to their farm data, and opting in or out of a service provider's system. A recent privacy invasion case was investigated by the Federal Trade Commission in USA on John Deere's data practices (Herchenroeder, 2021). This investigation found that John Deere does not consider basic privacy rights for farmers. For instance, John Deere is not giving farmers appropriate choices in terms of data access and collection of their farm data. Data collection cannot be halted at any time by the farmer and John Deere can use the data for any purpose they desire.

## Recommendations for agricultural technology providers

ATPs in the farming ecosystem are companies that build technologies and platforms for smart farming or install, integrate and govern technologies on farms (Huntington, 2020). In some ways, they can be considered as stewards of agriculture data and technology. Technology stewardship in agriculture refers to conducting, managing, or supervising the use of technology on farms (Gow, 2020). In many cases, ATPs even collect and control farm data, and as technology stewards, they should be held responsible for governance and management of data and farm technologies (Anidu and Dara, 2021). ATPs can also evaluate technology governance challenges such as security, sustainability, and privacy and provide advice to farmers on addressing these ethical concerns (Gow, 2020). This section provides recommendations for ATPs on how to establish ethical and responsible AI use in the farming and agriculture systems.

Transparency can be ensured through data license agreements. Since most privacy policies and terms of use agreements use legal terminologies, it can be difficult for farmers to fully understand the content of these documents (Kaur et al., 2018). The ambiguity of agreements and legal frameworks around data collection, processing, and sharing is another issue that may result in an invasion of farmers' privacy and can lead to misuse of data. These issues can be solved by ATPs by creating clear, complete, and unambiguous data and terms of use agreements. The data agreements discuss data practices and authorized use of technology in a simple language that is easy to understand for the farmers (Carbonell, 2016). In the agreements, ATPs should be clear and transparent about what data are collected from farmers and their farms, for what purpose data are used, and possibly even what data are used in building the AI models. Moreover, collected data should not be shared with third parties without farmers' consent (Kaur et al., 2018). Farmers should be given the choice to give or withdraw consent for the use of their data. Lastly, these agreements should elaborate on how ATPs address the issues related to data ownership and farmers' access to their data. Data agreements should also provide clear details on data retention, farm data security, cross-border data integration and transfer (if relevant) and other privacy and data confidentiality practices (Tsesis, 2019).

Transparency can also be enhanced through processes and technologies. Data access, portability, and harmonization are some of the recommended methods that can enhance transparency. These practices require that farmers should have the ability to download, access, use, and transfer their historical data to other farm management system suppliers. To achieve this, farmers' data should be available in a digital and well-structured, and machine-readable format. For example, an aviculture farmer may want to use a newer technology provided by another ATP to manage their poultry farm or build tools to monitor the health of their animal. Farmers may also want to share their farm data with farm co-ops to sell, integrate, or use their data for other purposes. To facilitate this as well as address data usability, and sustainability, ATPs should enable data portability for farm data. Data portability is the ability of users to move their data among different machines, systems, and applications. ATPs should also consider data interoperability and standardization while designing their platform in digital farming systems (Blobel et al., 2006). Interoperability is a system's ability to store, use, and exchange data in a uniform platform. Interoperability makes it possible to work with data in different domains and applications (Jaleel et al., 2020). Furthermore, interoperability can address robustness issues and is a requirement to comply with security protocols and enhance system security (Blobel et al., 2006).

Transparency in the use of AI approaches in agricultural technologies is critical in building trust with the farmers. Schwartz et al. (2022) Argue that most farmers are unaware of interacting with AI enabled technology (Blobel et al., 2006). In some other cases, it was reported that ATPs have obliged farmers to participate in farm data collection and use AI as a precondition to offer their service (Ryan, 2020). These are ethical dilemmas that ATPs should avoid. ATPs must inform farmers when an automated AI model is used in their farming system. Farmers also should be free to accept or deny the use of AI solutions or be the subject of AI decisions. This is a major ethical dilemma to tie the use of AI with the farmers' technology needs. Farmers should also have the right to ask about the outcome of AI tool, how the decision was made, what data were used, and what rules were extracted from data. This can be achieved by using interpretable and explainable AI methods (Rai, 2020). Interpretability and explainability of AI models, if presented in a user-friendly manner, can increase farmers' trust in the digital agricultural system (Rai, 2020).

Requirements to ensure robustness can impact the design of the AI-based technology, deployment and usage processes, as well as protocols. In many cases, farmers are not comfortable working with new technologies because of tools' complexity or farmers' unfamiliarity with the technology. ATPs should provide training sessions for farmworkers to help them gain the right skills with all hardware and software AI tools in the technology. Also, including farmers in the process of designing and developing AI-based decision technologies and in validating and testing these tools leads to more usable and trustworthy systems. In addition, farmers should be invited to take part in user studies and usability testing of the technology to ensure the usefulness and enhance user-friendliness of the tools, and improve their comfort level with using the technologies. Brochures and guidelines should be created for farmers on how to operate AI-based technologies. These resources should be created in a straightforward and understandable way so that the farmers are encouraged to use the resources and the technologies. These practices may result in more equitable and fair treatment for all farmers or stakeholders in the agriculture supply chain.

Reliability is one of the requirements that satisfy robustness. The usability and reliability of AI technology in farms are highly related to the accuracy of recommendations made by AI-based systems (Barocas and Selbst, 2016). Inaccurate outcomes provided by the technology tools lead to accountability problems. If the system has the wrong interpretation or recommendation on soil nutrition, for example, it can use improper fertilizers which in turn will affect the farm product and result in financial losses. To ensure reliability, it is required to pay attention to data quality that is used to build the AI model to avoid errors and biases (Jaleel et al., 2020). ATPs should monitor data collection and processing to detect data quality issues and reduce the impact. Furthermore, to achieve accurate predictions and recommendations, appropriate AI models should be used based on the available data and the problem objectives. Some models are data-intensive and require a large amount of data to operate accurately and reliably. For these models, high-quality and diverse data are usually needed to enable them to learn about diverse conditions and generalize well.

Privacy concerns in a farm system should be addressed by the ATPs. It is recommended that ATPs should follow the privacy by design (PbD) recommendations while designing a platform for digital farming or working with large-scale connected farm data systems (Cavoukian et al., 2009). PbD provides instruction on creating effective approaches to integrating data protection while designing a system. PbD promotes foundational principles that ATPs should consider for designing the digital agriculture platforms (Raji et al., 2020) such as end-to-end security and full transparency with the end-users. Another example of PbD principles is privacy as default. In farm technologies, de-identification at the source can enable default privacy and address identifiability concerns before storing or sharing farm data (Joo et al., 2018). This approach separates personal information from records stored in the data tables and stores them in other locations (Raji et al., 2020). Pseudonymization (Hintze and el Emam, 2018) is also a preventive solution to protect from data identifiability (Sedayao et al., 2014). Pseudonymization replaces features that could be used to identify a person with a value that does not imply a person's identification directly. Substituting farmers' names with an identification code is a simple example of pseudonymization to store their data. To address identifiability issues, ATPs can disable location data collection for sensors and devices while setting technologies up at the farm. ATPs should also consider performing a privacy impact assessment (PIA). PIA is a tool with which the potential privacy risks are identified and addressed while installing farm equipment and setting up data collection procedures. Furthermore, explicit consent must be obtained from the farmers before collection or processing or sharing of data. Other privacy solutions can include enabling opt-in or opt-out of data agreements, limiting data collection, and deleting data on request. Data protection impact assessments could be used to assess privacy concerns related to an AI system. This way, privacy risks and rights imposed by AI system can be proactively identified and managed.

Bias and discrimination by AI tools affect fairness. Building AI tools that favor organizations and actors in power, grant economic or social privileges to a particular group, or create power imbalance due to access and control of farm data are all ethical dilemmas that ATP providers should collaboratively address with farmer organizations, government, and other agencies (Panch et al., 2019). Bias in AI models and their use may be caused intentionally or unintentionally by those who created AI models or because of embedding pre-existing biases into the model. Collecting sufficient data from diverse sources, defining fair and ethical objectives for the use of data, and considering all members of society that are impacted by the AI systems and their outcomes can protectively eliminate presence of bias.

Many scientists believe that AI algorithms cannot address fairness concerns (Urruty et al., 2016). Risk of human rights invasion, fairness, and other fundamental ethical values that may be compromised by AI technologies are examples of ethical dilemmas in smart farming. Human-Centered AI (HCAI) can be a solution to mitigate these concerns. HCAI intersects AI with humanities by combining embedded AI support services with user experiences (Shneiderman, 2020). HCAI can bring ethical considerations and farm practices to AI models' performance in digital agriculture (UNESCO, 2021). Co-creating models and solutions and engaging all stakeholders as well as the government can lead to more ethical outcomes from HCAI (Camaréna, 2020). Also, since AI-based decisions are susceptible to bias and discriminatory outcomes, HCAI can manage to prevent these risks.

Issues related to the robustness of AI technologies may affect reliability and performance of the system. AI and automated systems are more susceptible to security attacks and require defense mechanisms that should be embedded in advance. In addition to attacks that may target digital and connected technologies, AI models and data can also be attacked by adversaries with the aim of detreating their performance. ATPs should take proactive measures to monitor data, system, network, and all other digital infrastructure in the farm continuously to reduce the possibility of cyber-attacks as much as possible. ATP should also monitor data breaches such as unauthorized access to farm data and data perturbations (Gupta et al., 2020). Using the risk assessment methods (Gupta et al., 2020) is also a common practice, including finding potential vulnerabilities and loopholes in the system by assessing potential risks in/to the farm system.

Robustness from a hardware perspective requires farm equipment to work properly and reliably (Urruty et al., 2016). For example, in the livestock industry and in big farms, milking dairy cows is performed every day by automatic equipment. If there is a sudden failure in technology, it is not feasible to milk hundreds of cattle by human labor. Such failures may threaten the animals' welfare and cause financial harm to farmers. To address this problem, AI models can in some cases predict when an asset or hardware may fail to assist farmers or ATPs to take proactive actions. For instance, such predictive tools can recommend when equipment can be serviced or replaced before failing. This can help in reducing unplanned downtime, increased safety, lower costs, extended asset life, and higher asset utilization rates. Failure and errors resulting from technologies' malfunction or cyberattacks may occur despite all types of measures and efforts to prevent them. Therefore, ATPs should present an incident response or failover recovery plan for such cases. This recommendation requires that ATPs proactively address probable issues such as system crashes or attacks to protect farm data and failures in the farm system (Shneiderman, 2020). Moreover, terms of use and data agreements should clearly specify how the organizations will be accountable and compensate for the damage caused by error, mismanagement, or other deficiency or failure in the system (Shneiderman, 2020).

There is a significant interest in developing AI tools that help ensure sustainable practices and are environmentally sustainable tools. AI and automated technologies may cause harm and distress to animal welfare, and negatively affect the environment. ATPs should consider design strategies that take into consideration the requirements for environmental sustainability. Milking robots should be developed in a way to avoid harming animals and causing pain. Greenhouse robots should not damage greens, crops or fruits. Factors affecting the environment and animals should always be considered while designing and deploying AI tools. Furthermore, ATPs can use fleet management technology in digital farming systems to decrease their carbon footprint. Fleet management uses the science of advanced systems to address sudden machine/vehicles' failure (Sørensen and Bochtis, 2010). These systems can estimate important metrics and indicators about farm equipment, such as fuel usage, engine speed, and upcoming maintenance, so that the system or ATPs can optimize the system's productivity and efficiency (e.g., fuel consumption) which can also help farmers save money.

Finally, tools and frameworks have been designed to detect and mitigate performance risks of AI models proactively and by design (TensorFlow, 2022). For example, there are tools (Google, 2022) and platforms (OECD AI, 2019) that can improve AI models' transparency. This way, farmers can understand the reasons for the decisions and combine their expertise with the results of the algorithms for a better outcome. ATPs can use platforms such as Responsible AI by Google (Google, 2022) and Explainable AI by IBM (IBM, 2002; Trustworthy AI, 2021) to develop and deploy AI solutions that are interpretable, less bias, and inclusive. Several Ethical AI frameworks, tools and standards such as IEEE Ethics in Action in Autonomous and Intelligent Systems (IEEE SA, 2022), ISO/IEC JTC 1/SC 42 Artificial intelligence (ISO, 2022), SCC's Ethical design and use of automated decision systems (Standards Council of Canada-Conseil canadien des norms, 2022), and European Commission's Ethics Guidelines for Trustworthy AI (European Commission, 2022) can be adopted to consider and address ethical requirements in the AI applications for farming. TEthical assessment of new agricultural and food technologies, such as ethical bio-technology assessment tools (Beekman et al., 2006) helps improve ethical consideration in all stages of the food chain. Using a responsible AI solution can strengthen trust between farmers and other actors in the value chain and increase adoption.

## Recommendations for policymakers

Governments can be instrumental in ensuring and enforcing ethical and responsible innovation in digital agriculture. Governments can establish regulations, guidelines, or best practices to guide technology providers and other actors in the value chain to develop and use digital tools and platforms that are for the greater good of communities and society. Tools and protocols can be developed or promoted to validate ethical principles, such as fairness, explainability, and privacy (ongov/Transparency-Guidelines, 2022). Also, governments can enforce stakeholders to use frameworks and assessment methods to evaluate the ethical aspects of AI tools before and after deployment in digital agriculture.

Policymakers can play an important role in providing a higher level of understandability of AI based technologies for farmers and agriculture actors (Pylianidis et al., 2021). Policymakers can help in educating farmers with AI skills that can help them understand how AI models and robotic technologies work. Policymakers should also take appropriate steps, through social dialogue or other means, to ensure a fair transition for farmworkers and other stakeholders to adopt technology that can transform the digital agricultural system. Furthermore, policymakers should also work closely with other stakeholders and promote responsible use of AI at work to fairly share the AI benefits and foster the entrepreneurship and productivity of digital farming systems.

The safety of AI equipment can also be a concern similar to many other technologies. The only difference is that the concerns in the AI system could be related to data, AI model's ability to generalize, or cyber-attacks on AI systems. Nevertheless, the safety concerns of AI systems can be partly mitigated by education and training for the users of these tools. The education part can be according to workplace safety issues, such as working with autonomous vehicles on the farm and greenhouse facilities to protect farm workers from risks in their workplace. When farm workers' safety is compromised with AI technologies, insurance coverage should be in place to compensate and help them in alleviating the consequences. Education programs can be prepared for ATPs and farmworkers on protecting the environment and reducing emissions, such as leaking toxic chemicals on the farm. Animal welfare is another ethical issue that requires attention in the context of new agricultural innovations, such as using automatic milking equipment for cattle. Government can provide incentives or funding for AI tools that are built and extensively tested to protect animal welfare and ensure environmental sustainability.

Neglecting farmers' privacy, lack of transparency and ambiguity in accountability may affect farmers' trust. This can lead to farmers' reluctance to acquire and use AI technologies and collaborate in data sharing activities. Policymakers can help mitigate some of these concerns by sponsoring development of innovative AI solutions that are trustworthy and by creating policy and regulatory frameworks and assessment mechanisms to test and validate trustworthiness of AI systems. Also, policymakers can consider creating and enforcing policies on issues related to accountability for AI-based farm tools. If farmers experience financial or reputation loss, ATPs or other stakeholders (e.g., farm insurance) should compensate the farmers. Establishing new policies or expanding existing policies to protect farmers' sensitive data, and their privacy, and enforcing transparency are important steps to ensure ethical and responsible use of these technologies.

Fair opportunities to access, use and take advantage of AI-based technologies for small and medium size farmers are important. AI technologies are generally targeted or priced for large farms which can widen the inequalities or digital divide for small-scale farmers (ACM Interactions, 2022). To improve fairness and create a sense of inclusion for small and medium farmers, governments should foster the development of a digital ecosystem for AI technologies so that the technologies are accessible and targeted to all farming stakeholders (OECD AI, 2019). Furthermore, governments can support and promote digital infrastructures for shared data processing such as cloud services and edge computing and communication technologies such as satellite, Wi-Fi, and 5G cellular (Threats to Precision Agriculture, 2018). These facilities can provide diverse farming systems with a trusted framework to share their data, process data in a secure manner, and share knowledge and insight while preserving privacy. These shared infrastructures have many benefits including facilitating data and technology interoperability, inclusivity, fairness for access to data and technologies as well as food safety and sustainability.

Policymakers also have a key role in supporting research and development (R&D) for ethical and responsible AI innovation for digital agriculture (OECD AI, 2019). Long-term investment in R&D, such as inter-disciplinary efforts to encourage human-centered AI and to address legal, social, and ethical implications, can encourage various actors in agriculture to collaborate on building trustworthy AI technologies for farming and food supply chain. Investment in open data repositories to protect farm data and provide an equitable environment to access data can also support collaboration and development of human-centered AI in farming. Open data repositories can improve interoperability and use of standards in digital agricultural systems which can help with development of system's robustness.

Although R&D is a necessary step to improve AI systems for farming, deployment and adoption of these technologies are still a challenge. Governments can establish an innovation network or platform for an agile transition from R&D to the deployment and operation stage (OECD AI, 2019). An operational practice to deploy the model is using experimentation (e.g., experimental farms) to provide a controlled environment to test and scale up the AI system. Governments can support experiments in “start-up mode” for the AI technologies to be deployed, evaluated, and modified and consequently help scale up if they pass ethical and performance requirements. Governments can also support general pilot projects, living labs, and digital twin initiatives in experimentation farms to develop controlled and transparent environments.

Educating farmers and farm workforces on various aspects of ethical AI technologies is also important. International cooperation for trustworthy AI can improve ethics on a broader level. It is recommended that governments or NGOs co-operate across borders and sectors, specifically in developing countries, and work toward responsible stewardship of AI by developing standards and exchanging information. Cross-border collaboration can lead to advancing societal goals, democratic values, and human rights and foster the sharing of AI knowledge as recommended by OECD (OECD AI, 2019). Furthermore, policymakers should also encourage international metrics to measure AI R&D and deployment to enhance implementation of ethical AI principles. Using ethical metrics can help assess the AI systems' performance, such as efficiency, accuracy, robustness, and fairness.

Farming practices can damage the environment by generating greenhouse gases such as carbon dioxide and nitrogen dioxide. There is a need to promote sustainable agriculture tools so that the natural resources and ecology are preserved. Therefore, the government should motivate farmers to use green technologies and renewable energies to slow down climate change and address sustainability concerns. Since the required equipment for embedding renewable technologies is too expensive, many farmers cannot afford to equip their farms. Governments' support is fundamental for farmers in providing the required knowledge and equipment. Also, policymakers can establish rules, such as tax exemption, to support farmers interested in employing green technologies to save the environment.

## Conclusion

This paper reviewed the ethical challenges in the digital agriculture domain and proposed an ethical framework which could be used when designing and deploying AI based technologies in farming. The paper also provides recommendations for ATPs and policymakers on the issues and potential solutions that can be considered for ethical and responsible AI in agriculture. The presented ethical AI framework helps in investigating the ethical principles and consequences of neglecting each principle in the agricultural ecosystem. AI ethical framework included principles such as fairness, transparency, accountability, sustainability, privacy, and robustness in the context of agriculture.

The recommendation for ATPs and policymakers included social, ethical, technology, and political considerations. ATPs are encouraged to perform risk assessments for ethical implications of AI systems under development and consider requirements and strategies that can avoid harmful consequences. These organizations should also collaborate with various actors in the value chain to collectively address ethical issues that may arise from the use of these technologies. Best practices and protocols such as transparency, providing technology functionalities that enable data portability, interpretability and usability, HCAI, raising awareness, and training opportunities for farmers were only some of the recommended practices in this paper. Recommendations for policymakers included creating new policies or extending existing policies for farm data and infrastructure, funding R&D initiatives, supporting living lab initiatives, and providing educational programs for farmers and other actors in the smart farming system.

Farmers are the main stakeholders in the agricultural ecosystem. As the end users of AI technologies, they can take a few steps to protect their farms, farm data, machinery, and farmworkers. Farmers can keep themselves up to date about AI technologies that are being used at their farms and how they operate. They should review the data and terms of use agreements carefully to understand what data is collected from their farm, with whom their data is shared, and how accountability is integrated into agreement and management. They should also inquire from ATP whether an AI model is embedded in their machines, what data the AI system uses, and what indicators/factors are extracted from the data by the AI system. Farmers should secure their machines from unauthorized access and use, and physically secure them when they are not in use. They should demand transparency regarding data use and their rights to data access and portability.

Researchers can also play a critical role in the development, validation, and governance of ethical and responsible AI systems for agriculture. Researchers can be instrumental in building trust between ATPs and farmers. They can conduct qualitative and quantitative research to understand farmers' needs and concerns, ethical dilemmas, AI technology requirements and bridge the knowledge gap between different stakeholders. New technologies, algorithms, and best practices can be proposed for agricultural AI-based systems to ensure fairness, robustness and reliability, transparency, sustainability and other ethical considerations. Researchers can work closely with government, ATPs, and farmers on new policies and standards to promote responsible innovation in digital agriculture and protect stakeholders' interests.

There are still many unsolved issues that need to be addressed. For example, legal transparency in the context of smart farming is unclear. There is a need to develop and test best practices and protocols for transparency to enable ethical use of data and digital infrastructure and maintain utility of data. Fair use of AI system and data in digital agriculture is also not well defined. For instance, what constitutes an unfair use of AI technologies in farming or what business model can be established to make the outcome fair for all the actors. Accountability is a complex legal and technical issue. There is an urgent need for standardized practices and policies to find solutions and common agreements on these issues. This can be achieved through collaboration and knowledge transfer and sharing. For instance, licensed insurance agencies and farm co-ops can be given a more active role in ensuring transparency, protecting farmers' privacy, enabling fair use of farm data and infrastructure, and facilitating governance models for accountability and sustainability.

## Author contributions

RD and SH contributed to conception of the study. All authors have contributed to writing the paper, contributed to manuscript revision, read, and approved the submitted version.

## Funding

This research was funded by a Natural Sciences and Engineering Research Council of Canada (NSERC) Discovery Grant and Ontario Ministry of Agriculture Food and Rural Affairs, New Directions, funding awarded to RD.

## Conflict of interest

The authors declare that the research was conducted in the absence of any commercial or financial relationships that could be construed as a potential conflict of interest.

## Publisher's note

All claims expressed in this article are solely those of the authors and do not necessarily represent those of their affiliated organizations, or those of the publisher, the editors and the reviewers. Any product that may be evaluated in this article, or claim that may be made by its manufacturer, is not guaranteed or endorsed by the publisher.
